# *Smartphones* : A New Source of Pain in the Wrist and Hand in the 21 ^st^ Century – Letter Regarding the Study by Gonçalves et al.

**DOI:** 10.1055/s-0044-1787764

**Published:** 2024-07-11

**Authors:** Sitanshu Barik, Vishal Kumar, Vikash Raj

**Affiliations:** 1 All India Institute of Medical Sciences, Nagpur, Índia; 2 Postgraduate Institute of Medical Education and Research (PGIMER), Chandigarh, Índia; 3 All India Institute of Medical Sciences, Deoghar, Índia

Smartphones have become a part and parcel of the daily existence in our lives due to their multifunctional usefulness apart from normal phone features, which includes internet browsing, electronic mail, location services, camera, as well as third-party applications called “apps”, which let the user perform a whole gamut of activities. 1 With this premise, the study by Gonçalves et al. 2 was read and analyzed with interest, and it aimed to investigate the long-term use of smartphones as a risk factor for the development of morbidities in the wrist and fingers. The study concluded that there is a significant correlation between the length of smartphone use and discomfort in the wrist and fingers.

The study included 100 subjects with a history of smartphone use for the past 5 years. Considering the prevalence of smartphone use, which is reported to be of approximately 79% of the population in the age group between 18 and 44 years, an adequate sample size in a prospective study like this may have yielded more interesting results with greater validity. 3

The study used the Boston Carpal Tunnel Questionnaire (BCTQ) to assess the symptoms as well as the functional deficit in the included subjects. This patient-reported outcome measure (PROM) has been criticized since its functional scale does not cover activities related to computers or smartphones. 4 Since the study in question deals with the effects of smartphone use, the application of the BCTQ may have been inappropriate. The use of other validated tools, such as the Modified BCTQ, which contains two additional items related to electronic gadgets and driving, would have provided more valid results.

Studies with an adequate sample size calculated prospectively based on the prevalence rate of smartphone use along with the use of a PROM which encompasses the items related to smartphone use shall help us more in understanding the role of smartphones in the development of pain in the upper limb.

## References

[1] Choi J S Yi B Park J H The uses of the smartphone for doctors: an empirical study from samsung medical center Healthc Inform Res 2011 17 02 131 138 21886874 10.4258/hir.2011.17.2.131 PMC3155170

[2] Gonçalves A MDS Carmo V JGD Araújo L MC Pereira T MM Use of Smartphones as a Risk Factor for the Development of Morbidities in the Wrist and Fingers Rev Bras Ortop 2023 58 03 457 462 10.1055/s-0042-1748948 PMC10310410 37396082

[3] Mustafaoglu R Yasaci Z Zirek E Griffiths M D Ozdincler A R The relationship between smartphone addiction and musculoskeletal pain prevalence among young population: a cross-sectional study Korean J Pain 2021 34 01 72 81 33380570 10.3344/kjp.2021.34.1.72 PMC7783853

[4] Miedany Y PROMs for Carpal Tunnel Syndrome 2016:329–355 [cited 2023 Sep 17]. Available from:10.1007/978-3-319-32851-5_13

