# Supplementary material for: Smartphones : A New Source of Pain in the Wrist and Hand in the 21 st Century – Letter Regarding the Study by Gonçalves et al
Source: Rev Bras Ortop (Sao Paulo). 2024 Jul 11;59(5):e805. doi: 10.1055/s-0044-1787764 (PMC11624918; doi:10.1055/s-0044-1787764)
Supplement: Related texts

# Research article: Smartphones: Uma nova fonte de dor no punho e na mão no século XXI – Carta sobre o estudo de Gonçalves et al.

Os *smartphones* tornaram-se parte integrante da existência diária em nossas vidas devido à sua utilidade multifuncional que vão além dos recursos normais do telefone, como navegação na internet, correio eletrônico, serviços de localização, câmera e aplicativos de terceiros, chamados *apps* , que permitem ao usuário realizar toda uma gama de atividades. 1 Com essa premissa, lemos e analisamos com interesse o estudo de Gonçalves et al., 2 cujo objetivo foi investigar o uso prolongado de *smartphones* como fator de risco para o desenvolvimento de morbidades no punho e nos dedos. O estudo concluiu que existe uma correlação significativa entre o tempo de uso do *smartphone* e o incômodo no punho e nos dedos.

O estudo incluiu 100 indivíduos com histórico de uso de *smartphones* nos últimos 5 anos. Considerando a prevalência do uso de *smartphones* , que gira em torno de 79% da população na faixa etária de 18 a 44 anos, um tamanho de amostra adequado em um estudo prospectivo como este poderia ter gerado resultados mais interessantes e com maior validade. 3

O estudo utilizou o Boston Carpal Tunnel Questionnaire (BCTQ) para avaliar os sintomas e o déficit funcional nos indivíduos incluídos. Esta medida de resultados relatados pelo paciente ( *patient-reported outcome measure* , PROM, em inglês) tem sido criticada, uma vez que a sua escala funcional não abarca atividades relacionadas a computadores ou *smartphones* . 4 Como o estudo em questão trata dos efeitos do uso do *smartphone* , a aplicação do BCTQ pode ter sido inadequada. A utilização de outra ferramenta validada, como o BCTQ Modificado, que conta com dois itens adicionais relacionados a aparelhos eletrônicos e direção, teria fornecido resultados mais válidos.

Um estudo com tamanho de amostra adequado, calculado prospectivamente com base na taxa de prevalência do uso de *smartphones* juntamente com o uso de PROM que englobe os itens relacionados ao uso destes dispositivos nos ajudará mais na compreensão do papel dos *smartphones* no desenvolvimento de dor no membro superior.
